# CLCA4 inhibits cell proliferation and invasion of hepatocellular carcinoma by suppressing epithelial-mesenchymal transition via PI3K/AKT signaling

**DOI:** 10.18632/aging.101571

**Published:** 2018-10-11

**Authors:** Zhao Liu, Mi Chen, Lin-Ka Xie, Ting Liu, Zhen-Wei Zou, Yong Li, Peng Chen, Xin Peng, Charlie Ma, Wen-Jie Zhang, Pin-Dong Li

**Affiliations:** 1 Cancer Center, Union Hospital, Tongji Medical College, Huazhong University of Science and Technology, Wuhan 430022, China; 2 Institute of Infection and Immunology, Union Hospital, Tongji Medical College, Huazhong University of Science and Technology, Wuhan 430022, China; 3 Department of Oncology, Renmin Hospital, Hubei University of Medicine, Shiyan 442000, China; 4 Department of Radiation Oncology, Fox Chase Cancer Center, Philadelphia, PA 19111, USA; 5 Department of Pathology, Shihezi University School of Medicine, Shihezi, Xinjiang 832002, China

**Keywords:** hepatocellular carcinoma, CLCA4, EMT, PI3K/AKT, prognosis

## Abstract

Calcium activated Chloride Channel A4 (CLCA4), as a tumor suppressor, was reported to contribute to the progression of several malignant tumors, yet little is known about the significance of CLCA4 in invasion and prognosis of hepatocellular carcinoma (HCC). CLCA4 expression was negatively correlated with tumor size, vascular invasion and TNM stage. Kaplan-Meier analysis showed that CLCA4 was an independent predictor for overall survival (OS) and time to recurrence (TTR). In addition, CLCA4 status could act as prognostic predictor in different risk of subgroups. Moreover, combination of CLCA4 and serum AFP could be a potential predictor for survival in HCC patients. Furthermore, CLCA4 may inhibit cell migration and invasion by suppressing epithelial-mesenchymal transition (EMT) via PI3K/ATK signaling. Knockdown of CLCA4 significantly increased the migration and invasion of HCC cells and changed the expression pattern of EMT markers and PI3K/AKT phosphorylation. An opposite expression pattern of EMT markers and PI3K/AKT phosphorylation was observed in CLCA4-transfected cells. Additionally, immunohistochemistry and RT-PCR results further confirmed this correlation. Taken together, CLCA4 contributes to migration and invasion by suppressing EMT via PI3K/ATK signaling and predicts favourable prognosis of HCC. CLCA4/AFP expression may help to distinguish different risks of HCC patients after hepatectomy.

## Introduction

Hepatocellular carcinoma (HCC) is one of the commonest malignant tumors and a leading cause of cancer-related death [1,2]. The overall survival of HCC patients remains unsatisfactory because of a high incidence of recurrence and metastasis after hepatic resection [3,4]. However, the promising therapy for HCC metastasis is not available. Understanding the molecular mechanisms underlying HCC metastasis is of crucial significance to development of therapeutic strategies for advanced HCC patients. Although several prognostic factors have been reported in HCC [5,6] [REMOVED HYPERLINK FIELD], there still lacks of suitable biomarkers that can be widely used in clinical settings [7,8].

Calcium activated Chloride Channel (CLCA) regulators are a family of proteins that are characterized as a symmetrical multiple cysteine motif in aminoterminal tail [9]. It has been reported that the human CLCA gene is positioned on chromosome 1p31-1p22 [10]. CLCA proteins play an important role in chloride conductance in epithelial cells [11]. Previous studies showed that CLCA members were involved in cell differentiation, apoptosis, adhesion and the progression of inflammatory in the airway [12]. In addition, more and more evidence found that the expression of CLCA proteins was abnormal in a variety of cancers, such as CLCA1, CLCA2 and CLCA4, which could be potential predictors for patients. CLCA1 could inhibit the proliferation of colorectal cancer cells, which is correlated with beneficial prognosis of colorectal cancer patients [13]. CLCA2 is a p53-inducible inhibitor of cell proliferation, and could be a marker of differentiated epithelium that is downregulated with tumor progression. Low CLCA2 expression promoted cell proliferation and metastasis which was caused by driving epithelial to mesenchymal transition (EMT) signaling pathway [14,15].

CLCA4 is a member of the CLCA family, which has similar primary structure with CLCA1 and CLCA2 [16]. It has been reported that high expression of CLCA4 was found in human brain, small intestine, lung and colon tissues [12]. On the contrary, loss of CLCA4 expression was observed in breast and bladder cancer that facilitated tumor cells growth and metastasis by the way of EMT [17,18]. However, the role and mechanism of CLCA4 in prognosis of HCC patients has not been well clarified.

In this study, we tested the expression of CLCA4 in HCC tissues and cells, and explored the association of CLCA4 expression with clinicopathological features and prognosis in HCC patients. EMT is a key process in cancer metastasis by which tumor cells acquire migratory characteristics, thereby disassociating from the primary tumor and migrating to distant sites. We further evaluated the correlation of CLCA4 expression with EMT markers in HCC tissues and cells. Moreover, high AFP level predicts unfavourable prognosis for HCC patients [19]. Therefore, we also analyzed the prognosis value of CLCA4 expression combined with serum AFP level in HCC patients.

## RESULTS

### Correlation of CLCA4 expression and clinicopathological characteristics

**Figure 1:**
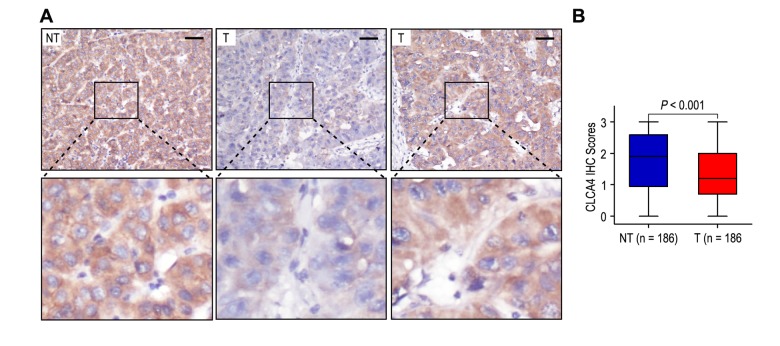
**The expression of CLCA4 was downregulated in hepatocellular carcinoma tissues.** (**A**) Immunohistochemistry assays of CLCA4 expression in HCC samples and adjacent non-tumorous tissues. The upper left panel represents high CLCA4 expression in adjacent non-tumorous tissues. The upper middle and right panel represents low and high CLCA4 expression in HCC tissues. Lower panels represent magnified pictures of boxed area in the corresponding upper panels. The line scale bar represents 50 μm. (**B**) CLCA4 expression in HCC tissues was compared with that in adjacent non-tumorous samples. Statistical analysis was performed by Paired-Samples *t*-test.

In order to explore the clinical significance of CLCA4 in HCC patients, we detected the expression of CLCA4 in 186 HCC tissues and their matched adjacent non-tumorous liver samples by immunohistochemistry. Classical photos showed that CLCA4 located in cytoplasm of HCC cells (Fig. 1A). The incidence of CLCA4 low expression in HCC samples was 55.9% (104/186), while there was 33.9% (63/186) in the matched adjacent non-tumorous liver samples. The expression of CLCA4 was decreased in HCC tissues (*P* < 0.001; Fig. 1B).

We further analyzed the correlation of CLCA4 expression with clinicopathological factors in 186 HCC samples. The expression of CLCA4 was negatively correlated with tumor size (*P* = 0.030), vascular invasion (*P* = 0.004) and TNM stage (*P* = 0.044) (Table 1). On the contrary, CLCA4 expression had no significance with gender, age, AFP level, HBsAg, gamma-glutamyltransferase (GGT), liver cirrhosis, tumor number, satellite nodule, tumor differentiation and BCLC stage (all *P* > 0.05).

**Table 1:** Correlation of CLCA4 protein expression with clinicopathological parameters.

Characteristics	No. of patients	CLCA4 expression (%)		*P*-value
		Low	High	
Gender				
Female	22	9 (41.0%)	13 (59.0%)	0.131
Male	164	95 (57.9%)	69 (42.1%)	
Age (years)				
≤ 50	97	57 (58.8%)	40 (41.2%)	0.414
> 50	89	47 (52.8%)	42 (47.2%)	
AFP (ng/ml)				
≤ 400	110	55 (50.0%)	55 (50.0%)	0.051
> 400	76	49 (64.5%)	27 (35.5%)	
HBsAg				
Negative	12	7 (58.3%)	5 (41.7%)	0.861
Positive	174	97 (55.7%)	77 (44.3%)	
GGT (U/l)				
≤ 50	101	50 (49.5%)	51 (50.5%)	0.055
> 50	85	54 (63.5%)	31 (36.5%)	
Liver cirrhosis				
No	42	20 (47.6%)	22 (52.4%)	0.218
Yes	144	84 (58.3%)	60 (41.7%)	
Tumor size (cm)				
≤ 5	106	52 (49.1%)	54 (50.9%)	0.030
> 5	80	52 (65.0%)	28 (35.0%)	
Tumor number				
Single	172	98 (57.0%)	74 (43.0%)	0.306
Multiple	14	6 (42.9%)	8 (57.1%)	
Satellite nodule				
No	163	88 (54.0%)	75 (46.0%)	0.159
Yes	23	16 (70.0%)	7 (30.0%)	
Tumor differentiation				
I-II	128	73 (57.0%)	55 (43.0%)	0.648
III-IV	58	31 (53.4%)	27 (46.6%)	
Vascular invasion				
No	156	80 (51.3%)	76 (48.7%)	0.004
Yes	30	24 (80.0%)	6 (20.0%)	
TNM stage				
I	147	82 (55.8%)	73 (44.2%)	0.044
II	7	1 (14.3%)	6 (85.7%)	
III	32	21 (65.6%)	11 (34.4%)	
BCLC stage				
0	17	6 (35.3%)	11 (64.7%)	0.252
A	84	46 (54.8%)	38 (45.2%)	
B	65	39 (60.0%)	26 (40.0%)	
C	20	13 (65.0%)	7 (35.0%)	

### Low CLCA4 expression indicated poor prognosis in HCC patients

To discuss the prognosis value of CLCA4 status in HCC patients, we analyzed univariate analysis of CLCA4 expression and clinicopathologic features for prognosis (OS and TTR). Kaplan-Meier analysis revealed that the patients with low CLCA4 expression (*P* < 0.001), high AFP level (*P* < 0.001), high GGT level (*P* = 0.017), liver cirrhosis (*P* = 0.007), larger tumor (*P* < 0.001) and vascular invasion (*P* < 0.001) had shorter OS time. In addition, low CLCA4 expression (*P* < 0.001), high AFP level (*P* = 0.004), high GGT level (*P* = 0.010), liver cirrhosis (*P* = 0.019), larger tumor (*P* < 0.001), satellite nodule (*P* = 0.002) and vascular invasion (*P* < 0.001) were unfavourable prognostic factors for TTR of HCC patients (Table 2).

**Table 2:** Univariate and multivariate analysis of CLCA4 associated with survival and recurrence in HCC patients.

Variables*	OS				TTR			
Univariate	Multivariate			Univariate	Multivariate		
*P*-value	*P*-value	HR	95% CI	*P*-value	*P*-value	HR	95% CI
Gender (Female vs. Male)	NS	NS			NS	NS		
Age, years (≤ 50 vs. > 50)	NS	NS			NS	NS		
AFP (ng/mL) (≤ 400 vs. > 400)	< 0.001	0.002	1.955	1.270-3.010	0.004	0.035	1.546	1.031-2.319
HBsAg (Negative vs. Positive)	NS	NS			NS	NS		
GGT (U/l) (≤ 50 vs. > 50)	0.017	NS			0.010	NS		
Liver cirrhosis (No vs. Yes)	0.007	0.014	2.241	1.179-4.261	0.019	NS		
Tumor size (cm) (≤ 5 vs. > 5)	< 0.001	0.038	1.669	1.029-2.708	< 0.001	0.034	1.698	1.041-2.771
Tumor number (Single vs. Multiple)	NS	NS			NS	NS		
Satellite nodule (No vs. Yes)	NS	NS			0.002	NS		
Tumor differentiation (I-II vs. III-IV)	NS	NS			NS	NS		
Vascular invasion (No vs. Yes)	< 0.001	< 0.001	4.067	2.450-6.752	< 0.001	< 0.001	5.295	3.089-9.078
CLCA4 (Low *versus* High)	< 0.001	0.008	0.528	0.328-0.849	< 0.001	0.005	0.542	0.353-0.832

*TNM stage and BCLC stage was combined with several clinical indexes such as tumor size, number and tumor thrombus; we did not enter the TNM stage and BCLC stage into multiple analysis with these indexes to avoid any bias in analysis.

GGT gamma-glutamyltransferase, AFP α-fetoprotein, OS overall survival, TTR time to recurrence, NS not significant, HR hazard ratio, CI confidential interval.

**Figure 2:**
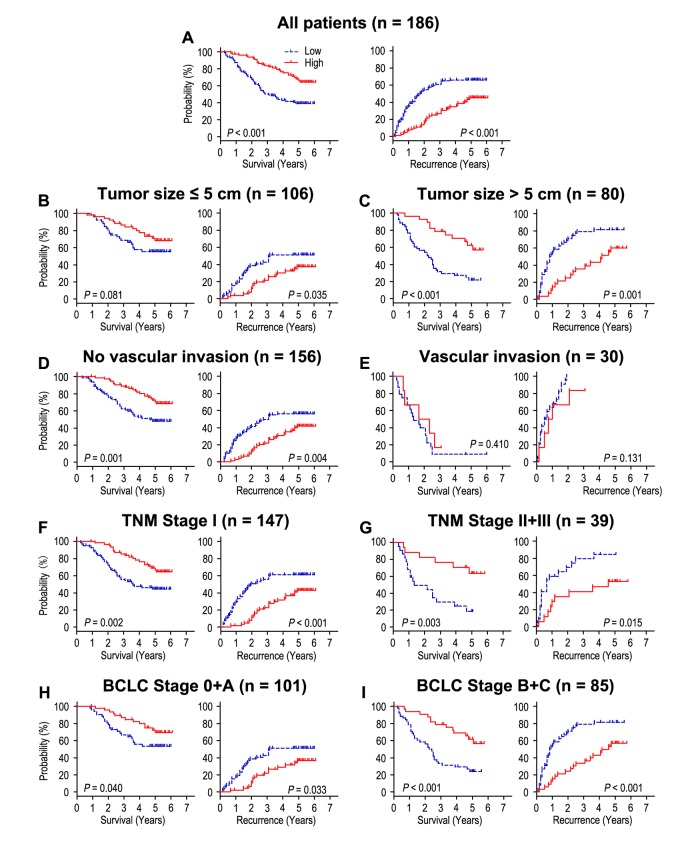
**The effect of CLCA4 expression on overall survival and time to recurrence is shown for patients with HCC.** All patients were classified according to tumor size, vascular invasion, TNM stage and BCLC stage. Kaplan-Meier survival estimates and log-rank tests were used to analyze the prognostic value of CLCA4 expression in all patients (**A**) and each subgroup (**B**-**I**).

Patients with high CLCA4 expression had better OS and TTR times than those with low CLCA4 expression (both *P* < 0.001) (Fig. 2A). In addition, the median of OS and TTR times in all the patients was 48.5 months and 34.0 months. The median of OS and TTR times in low CLCA4 expression group (n = 82) were 32.0 months and 20.5 months, while there were 62.0 months and 59.5 months in high CLCA4 expression group (n = 104). Moreover, the rates of 5-year OS and TTR of the low CLCA4 expression group were significantly lower than those of the high CLCA4 expression group (OS: 39.0% *vs*. 68.0%, TTR: 33.9% *vs*. 54.7%) (Fig. 2A). Furthermore, multivariable cox regression analyses revealed that low CLCA4 expression remained as an independent predictor in multivariate models and could be a risk factor for OS in HCC patients (HR = 0.528, 95% CI = 0.328-0.849, *P* = 0.008). The patients with low CLCA4 expression will be more likely to suffer from relapse than those with high CLCA4 expression (HR = 0.542, 95% CI = 0.353-0.832, *P* = 0.005), (Table 2).

To further explore the prognostic value of CLCA4 in different risk of subgroups, all the HCC patients were divided according to tumor size, vascular invasion, TNM stage and BCLC stage (Fig. 2B-I). Patients with low CLCA4 expression predicted poor OS and TTR times in all of these subgroups for except OS and TTR in patients who had vascular invasion (*P* = 0.410, and *P* = 0.131) or OS in patients with tumor size ≤ 5 cm (*P* = 0.081). In summary, our results indicated that CLCA4 expression could be used as prognostic predictor in different risk subgroups of HCC patients.

### Combined influence of CLCA4 and serum AFP on the risk of HCC prognosis

**Figure 3:**
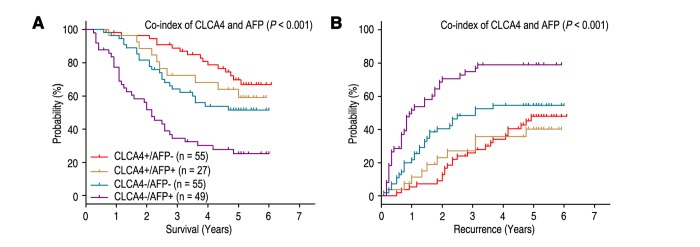
**Combined value of CLCA4 and serum AFP identify different risks of HCC death and recurrence.** The associations of CLCA4/AFP co-expression with Overall survival (log-rank *P* < 0.001) (A) and Time to recurrence (log-rank *P* < 0.001) (B) in 186 HCC patients.

Previous study showed that high serum AFP level was an unfavourable factor for the prognosis of HCC patients [19]. Our results revealed that patients with high serum AFP level (AFP > 400 ng/ml) had poorer OS (*P* < 0.001) and shorter TTR (*P* = 0.004), and could be an independent predictor for prognosis in HCC patients (Table 2). Furthermore, we combined the prognostic significance of CLCA4 expression and serum AFP levels to distinguish different prognosis (OS and TTR) of HCC patients. Based on the CLCA4 expression and serum AFP levels, all the HCC patients were divided into different risk subgroups: patients with CLCA4 (+) and AFP ≤ 400 ng/ml (CLCA4+/AFP-) had better OS time; patients with CLCA4 (+) and AFP > 400 ng/ml (CLCA4+/AFP+), or patients with CLCA4 (-) and AFP ≤ 400 ng/ml (CLCA4-/AFP-), had intermediate prognosis; patients with CLCA4 (-) and AFP > 400 ng/ml (CLCA4-/AFP+) were prone to death (Fig. 3A). Moreover, the patients with CLCA4+/AFP- or CLCA4+/AFP+ had longer TTR time; the CLCA4-/AFP- patients had intermediate-risk of relapse; while the CLCA4-/AFP+ patients were more likely to recurrence (Fig. 3B). Multivariate analysis revealed that co-index of CLCA4/AFP could act as an independent prognostic predictor for OS (HR = 1.224, 95% CI = 1.001-1.497, *P* = 0.049) and TTR (HR = 1.401, 95% CI = 1.167-1.682, *P* < 0.001) (Table 3).

**Table 3:** Univariate and multivariate analysis of co-index of CLCA4/AFP associated with survival and recurrence in HCC patients.

Variables	OS				TTR			
	Univariate	Multivariate			Univariate	Multivariate		
	*P*-value	*P*-value	HR	95% CI	*P*-value	*P*-value	HR	95% CI
Gender (Female vs. Male)	NS	NS			NS	NS		
Age, years (≤ 50 vs. > 50)	NS	NS			NS	NS		
HBsAg (Negative vs. Positive)	NS	NS			NS	NS		
GGT (U/l) (≤ 50 vs. > 50)	0.017	NS			0.010	NS		
Liver cirrhosis (No vs. Yes)	0.007	0.015	2.211	1.167-4.189	0.019	NS		
Tumor size (cm) (≤ 5 vs. > 5)	< 0.001	0.024	1.727	1.073-2.781	< 0.001	0.024	1.739	1.075-2.813
Tumor number (Single vs. Multiple)	NS	NS			NS	NS		
Satellite nodule (No vs. Yes)	NS	NS			0.002	NS		
Tumor differentiation (I-II vs. III-IV)	NS	NS			NS	NS		
Vascular invasion (No vs. Yes)	< 0.001	< 0.001	4.197	2.498-7.050	< 0.001	< 0.001	5.225	3.058-8.928
Co-index of CLCA4/AFP*	0.006	0.049	1.224	1.001-1.497	< 0.001	< 0.001	1.401	1.167-1.682

*Co-index of CLCA4/AFP was combined with CLCA4 and AFP, therefore, we did not enter the CLCA4 and AFP into univariate and multiple analysis with these indexes to avoid any bias in analysis.

GGT gamma-glutamyltransferase, AFP α-fetoprotein, OS overall survival, TTR time to recurrence, NS not significant, HR hazard ratio, CI confidential interval.

### CLCA4 inhibit cell migration and invasion in HCC

**Figure 4:**
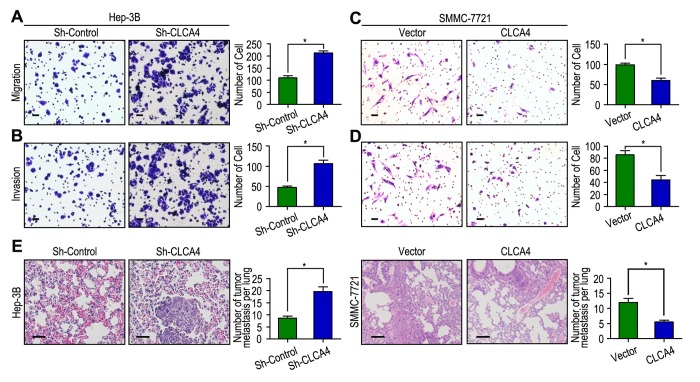
**CLCA4 inhibits cell migration and invasion in HCC cell lines.** (**A**, **B**) CLCA4 silenced in Hep-3B cells and promoted cell migration and invasion. (**C**, **D**) CLCA4 up-regulated in SMMC-7721 cells and inhibited cell migration and invasion. (**E**) Lung H&E staining of nude mice inoculated Hep-3B and SMMC-7721 transfected cells via tail vein. The number of lung tumor nodules in each group were also calculated, * *P* < 0.05.

We next detected the biological functions of CLCA4 in migration and invasion of HCC cell, which has been implicated from clinical data. The CCK-8 cell viability assays showed that knockdown of CLCA4 significantly promoted cell viability in Hep-3B cell line, while overexpression of CLCA4 had the opposite effect in SMMC-7721 cell line (Fig. S1). Moreover, IHC found that CLCA4 expression was negatively correlated with Ki-67 expression in HCC tissues (*r* = -0.410, *P* < 0.001, Fig. S2). Hep-3B cells migrated faster and had more ability to invade through Matrigel when CLCA4 gene was knocked down (Fig. 4A and B). In contrast, SMMC-7721 cells migrated slower and had less invasive ability when CLCA4 was upregulated (Fig. 4C and D). We further examined the *in vivo* effects of CLCA4 on tumor invasion in Hep-3B and SMMC-7721 xenografts *in vivo*. After 8 weeks, the mice were sacrificed, and the tumor nodules at the lung surfaces were counted. There were more numbers of tumor nodules on the lungs surface of mice when CLCA4 was silenced. On the contrary, with the SMMC-7721 cells, tumors developed from CLCA4-transfected cells were significantly smaller than tumors from vector cells. Hematoxyliin and eosin (H&E) staining confirmed that the nodules on the surfaces of mice lungs were tumors nodules (Fig. 4E). These data suggest that downregulated CLCA4 may promote the motile and invasive abilities of HCC cells.

### CLCA4 regulated HCC cell invasion via epithelial-mesenchymal transition (EMT) and PI3K/AKT signaling pathway

**Figure 5:**
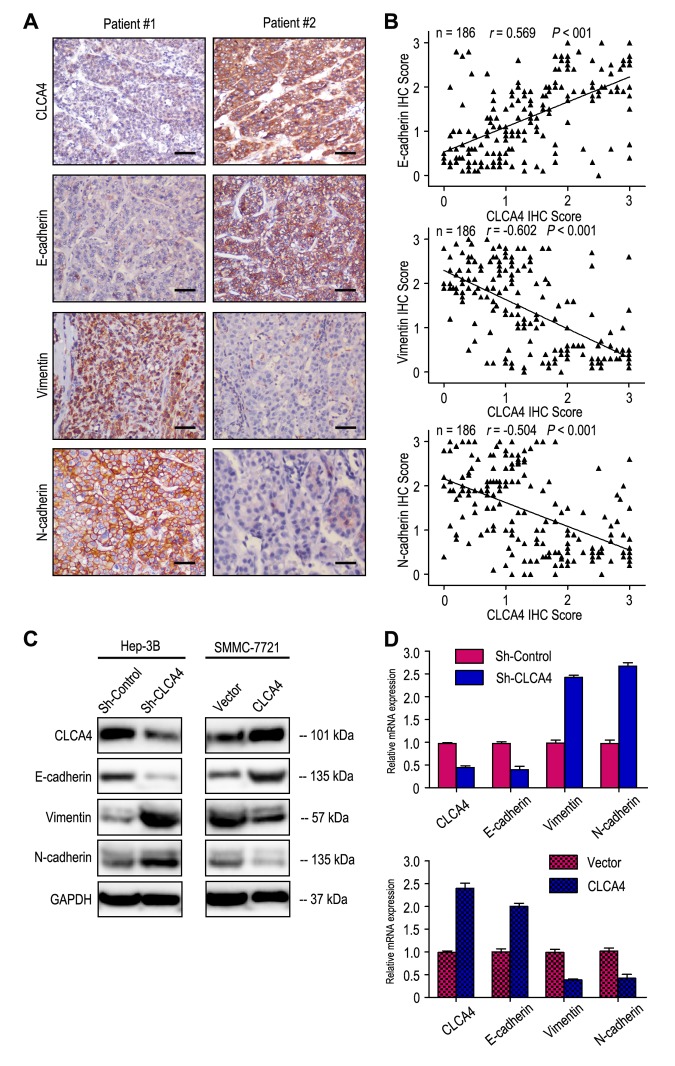
**CLCA4** **expression was correlated with the expression of epithelial-mesenchymal transition (EMT) markers.** (**A**) Serial sections of human HCC tissue were subjected to IHC staining with antibodies against CLCA4, E-cadherin, Vimentin and N-cadherin. In patient #1, low CLCA4 expression in HCC tissues was accompanied by the absence of E-cadherin and elevated Vimentin, N-cadherin. In patient #2, high CLCA4 expression was accompanied by elevated E-cadherin and the absence of Vimentin, N-cadherin. The scale bar represents 50 μm. (**B**) CLCA4 expression was positively associated with E-cadherin expression and negatively correlated with vimentin and N-cadherin expression. (**C**) Decreased expression of E-cadherin with increased expression of Vimentin and N-cadherin in CLCA4-silenced cells compared with the control cells. An opposite expression pattern of these genes was observed in CLCA4-transfected cells. (**D**) The mRNA of E-cadherin was down-regulated, while the vimentin and N-cadherin was up-regulated, when CLCA4 was silenced. And opposite expression was observed in CLCA4-transfected cells.

As Epithelial-mesenchymal transition (EMT) is one main process of tumor cell migration and invasion, we therefore evaluated the effect of CLCA4 on the expression of EMT-related markers. CLCA4 expression was positively related to E-cadherin expression and negatively correlated with Vimentin and N-cadherin expression in 186 HCC samples (Fig. 5A and B). Interestingly, down-regulation of E-cadherin and increased vimentin and N-cadherin expression were observed in CLCA4 silenced cancer cells. Conversely, up-regulation of E-cadherin and decreased vimentin and N-cadherin expression were detected in CLCA4 overexpressed SMMC-7721 cells (Fig. 5C). Like the results of IHC and western blotting, the RT-PCR results found that decreased expression of E-cadherin and increased expression of vimentin and N-cadherin in CLCA4-silenced cells compared with the control tumor cells, while opposite expression was observed in CLCA4-transfected cells (Fig. 5D). Together, our results suggest that CLCA4 may inhibit HCC cells migration and invasion by driving EMT pathway.

**Figure 6:**
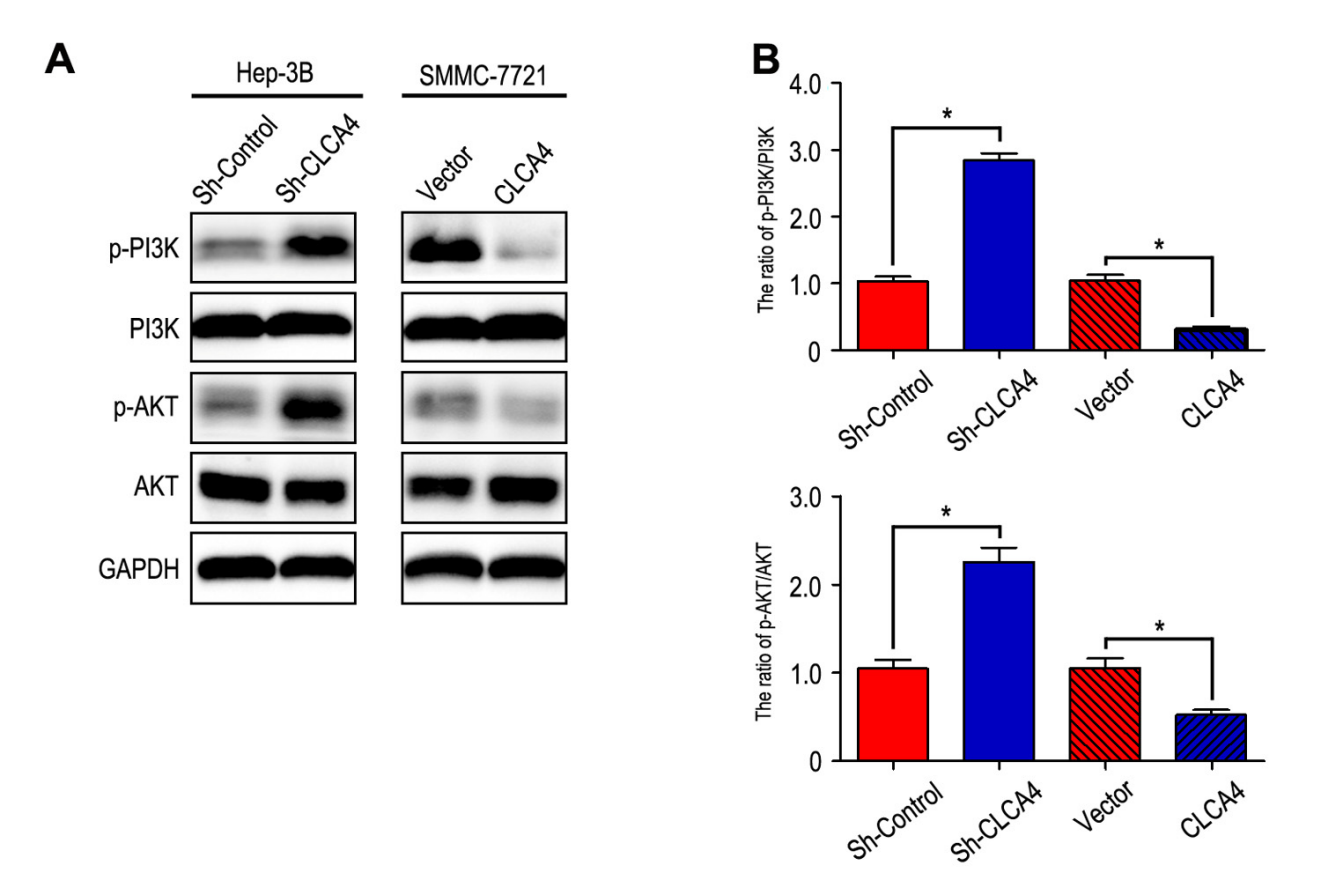
**CLCA4 inhibits HCC cell migration, invasion and EMT through suppressing PI3K/AKT signaling pathway.** (**A**) The levels of phosphorylated PI3K, total PI3K, phosphorylated AKT and total AKT were detected in HCC cell lines by western blot analysis. Increased expression of phosphorylated PI3K and phosphorylated AKT in CLCA4-silenced cells compared with the control cells. An opposite expression pattern of these genes was observed in CLCA4-transfected cells. Quantification of p-PI3K/PI3K and p-AKT/AKT was showed in (**B**).

It has been known that PI3K/AKT signaling pathway plays an important role in the development of cancer [20]. Therefore, we further investigated the effect of CLCA4 on the expression of certain molecules involved in the PI3K/AKT signaling pathway. Our results showed that phosphorylation of PI3K and AKT were enhanced in CLCA4-knockdown cells. On the contrary, overexpression of CLCA4 caused a significant decrease in PI3K and AKT phosphorylation (Fig. 6).

## DISCUSSION

It has been well established that CLCA4 plays an important role in carcinomatosis in several cancer types. Hou T et al. [18] reported that the expression of CLCA4 was lower in bladder carcinoma tissues than that in adjacent non-tumor tissues. Low CLCA4 expression was correlated with larger tumor size, advanced tumor stage, and poor prognosis in patients with bladder cancer. Yu Y et al. [17] found that CLCA4 was expressed in mammary epithelial cells and downregulated in breast tumors. Moreover, CLCA4 down-expression was associated with poor relapse-free survival in basal and luminal B breast cancers. However, the significance of invasion and prognosis of CLCA4 in HCC patients remains unknown. In this study, our results showed that the expression of CLCA4 in tumors was lower than that in adjacent non-tumorous tissues. In addition, CLCA4 expression was negatively correlated with tumor size, vascular invasion and TNM stage. Moreover, the Kaplan-Meier curves revealed that patients with low CLCA4 expression had poorer OS and shorter TTR times than those with high CLCA4 expression. Multivariate Cox regression analysis of the HCC patients found that low CLCA4 expression was an independent prognostic biomarker for HCC patients. Furthermore, the prognostic value of CLCA4 in different risk of subgroups according to tumor size, vascular invasion, TNM stage and BCLC stage was also analyzed, which found that CLCA4 could maintain its prognostic value in different risk of HCC patients for except OS and TTR in patients who had vascular invasion or OS in patients with tumor size ≤ 5 cm. Our results suggest that CLCA4 status is associated with tumor progression and could be used as prognostic factor for HCC patients.

It has been reported that serum AFP is a tumor marker that could be commonly used for diagnosis, predicting prognosis, and monitoring recurrence in HCC patients with high AFP level after resection [21,22] [REMOVED HYPERLINK FIELD]. However, it is difficult for clinician to predict survival and relapse for HCC patients who have normal AFP level after resection. To explore whether the prognostic value of CLCA4 expression combined with serum AFP level was superior to AFP alone, all the HCC patients were divided into four subgroups according to the CLCA4 expression and serum AFP level. Our study found that combination of CLCA4 expression and serum AFP level may be more conducive to distinguish different survival and recurrence of patients compared to CLCA4 expression or serum AFP level alone. In addition, co-index of CLCA4/AFP could act as an independent prognostic biomarker for HCC patients. Therefore, co-index of CLCA4/AFP to differentiate prognosis of patients may help to determine whether adjuvant therapy is required after resection.

Our results revealed that down-regulation of CLCA4 significantly promoted, while overexpression of CLCA4 suppressed HCC cell proliferation, migration and invasion. The biological function of CLCA4 in inhibiting tumor invasion was further supported *in vivo* experimental metastasis assay. It has been known that EMT, which is characterized by down-regulation of E-cadherin and up-regulation of N-cadherin and vimentin, is associated with enhanced cell motility, invasive phenotypes, and consequently the metastasis in human malignancies [23]. In HCC, E-cadherin, N-cadherin and vimentin are involved in various steps of metastasis, such as loss of cell-cell/cell-ECM adhesion, invasion of ECM, and angiogenesis [24,25] [REMOVED HYPERLINK FIELD]. Previous studies showed that knockdown of CLCA4 expression caused downregulation of E-cadherin and upregulated the expression of N-cadherin and vimentin in breast cancer cells [17].

It has been reported that PI3K/AKT signaling pathway plays an important role in cancer cell proliferation, migration and invasion [20,26,27]. PI3K is activated by oncogenes, which could promote tumor cell growth and survival [28]. AKT is a central signaling molecule in the PI3K pathway that is frequently activated in human cancer that stimulated the phosphorylation and impacted various downstream targets [20]. Moreover, the PI3K/AKT pathway is involved in the development and progression of HCC and is activated in 92.3% of HCC tissues [29,30]. In this study, we observed that down-regulation of CLCA4 obviously increased the expression of PI3K and AKT phosphorylation in HCC cells, while an opposite expression pattern of PI3K/AKT phosphorylation was detected in CLCA4-transfected cells. Therefore, CLCA4 inhibits HCC cell migration, invasion and EMT through suppressing PI3K/AKT signaling pathway.

## CONCLUSIONS

In general, the present study showed that CLCA4 may inhibit HCC cell migration and invasion by suppressing EMT via PI3K/AKT pathway, and could act as a potential prognostic biomarker for HCC patients. Moreover, the combination of CLCA4 expression with serum AFP level may be used to distinguish different risk of HCC patients after resection.

## MATERIALS AND METHODS

### Patients and specimens

The study was approved by the Institutional Review Board and Human Ethics Committee of Union Hospital, Tongji Medical College, Huazhong University of Science and Technology. Written informed consent was obtained from all the patients enrolled in this study.

All HCC samples were obtained from 186 consecutive patients who underwent hepatectomy from July 2010 to September 2011 at the Sun Yat-Sen University Cancer Center (Guangzhou, China). The inclusion criteria of this study were as follows: Firstly, all the patients did not have distant metastasis or received previous therapy before liver resection. Secondly, all the cases had been diagnosis for pathological proof. Lastly, there were no serious complications in perioperative period or no other malignant tumor. The clinicopathological features of HCC patients were showed in Table S1. Tumor stage was determined based on the 7th Edition tumor-node-metastasis (TNM) classification of the American Joint Committee on Cancer Staging and the Barcelona Clinic Liver Cancer (BCLC) staging system. Overall survival (OS) was computed as from date of operation to date of death or last follow-up. Time to recurrence (TTR) was defined from date of operation to date of recurrence or last follow-up.

### Cell culture

Two human HCC cell lines were used in this study: Hep-3B and SMMC-7721 cell lines. The two cell lines were obtained from Cell Bank of Shanghai Institutes for Biological Sciences, Chinese Academy of Sciences (Shanghai, China). Hep-3B and SMMC-7721 were maintained in DMEM supplemented with 10% fetal bovine serum and 100 units/ml penicillinstreptomycin (Invitrogen, USA) at 37°C in 5% CO_2_.

### IHC staining

All the 186 HCC samples and their matched adjacent non-tumorous liver tissues were detected by IHC method. Slides were baked at 55 °C for 1.5 h, deparaffinized with xylene and rehydrated using an alcohol gradient. The tissue slides were then treated with 3% hydrogen peroxide in methanol for 30 min to quench endogenous peroxidase activity, and the antigens were retrieved in 0.01 M sodium citrate buffer (pH 6.0) using a microwave oven. After antigen retrieval, the sections were incubated with 10% normal goat serum to block any nonspecific reaction, the sections were incubated using a primary antibody against CLCA4 (ab197347, Abcam, UK) or E-cadherin (ab1416, Abcam, UK) or Vimentin (ab92547, Abcam, UK) or N-cadherin (ab18203, Abcam, UK) or Ki-67 (ab15580, Abcam, UK), at 4 °C overnight. The tissue slides were treated with a non-biotin horseradish-peroxidase detection system according to the manufacturer’s instructions (Gene Tech). Immunohistochemical signals were scored by two independent investigators blinded to the patients’ identity and clinical status. In discrepant cases, a pathologist reviewed the cases, and a consensus was reached.

Both the extent and intensity of immunohistochemical staining were taken into consideration when analyzing the data. The intensity of staining was scored from 0 to 3, and the extent of staining was scored from 0% to 100%. The final quantitation of each staining was obtained by multiplying the two scores. CLCA4 expression was classified as high expression if the score was higher than the median score of 1.3, if the score was 1.3 or less, the case was classified as low expression.

### Plasmid constructs and transfection

cDNA containing open reading frames of CLCA4 was amplified by PCR and cloned into pcDNA3.1 vector (Invitrogen, Carlsbad, CA), and then transfected into SMMC-7721 cell using lipofectamine 2000 (Invitrogen) according to the manufacturer’s instructions. Cell transfected with empty vector was used as control. Stable CLCA4-expressing clones were selected by Geneticin (Rache Diagnostics, Indianapolis, IN) at the concentration of 500ug/ml.

### Establishment of CLCA4 knockdown cells

Short hairpin RNAs (shRNA) targeting the human CLCA4 gene was purchased from GeneRay Biotechnology (GeneRay Biotechnology Co., Ltd.) and transfected into Hep-3B cell using lipofectamine 2000 (Invitrogen) based on the manufacturer’s guidelines. Cell transfected with empty vector was used as control. Two days after transfection, cell culture media was collected and concentrated. Real-time PCR and Western blot were used to validate gene silencing efficiency of shRNA plasmids 48 hours after transfection. The recombinant lentivirus was stored at -80°C.

### Total RNA Extraction and qRT-PCR

Total cellular RNA was extracted from HCC cell lines using TRIzol reagent (Life Technologies, USA) based on the manufacturer’s instructions. RNA (2 µg) was reverse transcribed using a PrimeScript RT Kit (Takara, Japan). Real-time PCR was performed using SYBR master mix (Takara) on the Bio-Rad Connect Real-Time PCR platform. The primer sequences used for human CLCA4 were 5’-TTTGGGGCTCTTACATCAGG-3’ (forward) and 5’-GTGTCGTTCATCCAGGCATT-3’ (reverse). Relative mRNA quantities were determined using comparative cycle threshold methods and normalized against GAPDH.

### Western blotting

Total protein extracts were lysed in lysis buffer. The protein concentration was determined using a Bradford protein assay (Thermo Scientific, Massachusetts, USA). Equal amounts of protein were electrophoresed on SDS-PAGE and blotted onto PVDF membranes (Millipore Corp, Billerica, MA, USA). After the membranes were blocked by a 5% skim milk solution, the membranes were incubated overnight at 4°C with various primary antibodies (CLCA4, E-cadherin, Vimentin, N-cadherin or GAPDH from abcam, UK). The membranes were washed and incubated at room temperature for 2h with the secondary antibodies. Which were conjugated with horseradish peroxidase (HRP). The immunoreactive protein bands were visualized by ECL kit (Pierce, Rockford, USA).

### Cell counting kit-8 (CCK-8) assay

Cell viability was quantified by using the CCK-8 kit (DOJINDO Laboratories, Japan). Cells were plated on 96-well plates (0. 5×10^3^ cells/plate) and cultured for 5 days. Colonies were then fixed and stained with 1 mg/ml crystal violet.

### Cell migration and invasion assays

Cell migration and invasion assays were performed with BD BioCoat Matrigel Invasion Chambers (Becton Dickinson Labware, Franklin Lakes, NJ). After pretreated, Hep-3B and SMMC-7721 cells (5×10^4^ cells/ml) suspended in RPMI medium were added to the upper chamber. The lower chamber of the transwell was filled with 500μl DMEM containing 10% FBS as a chemoattractant. After 12h incubation, cells on the surface of upper chamber were removed by scraping with a cotton swab. The matrigel membrane was stained with crystal violet, and migrated/invaded cells were counted under a microscope.

### Experimental metastasis assay

All animal experiments were performed in accordance with NIH guidelines for the use of experimental animals. Male athymic BALB/c nude mice were purchased from the Shanghai Experimental Animal Center of Chinese Academic of Sciences (Shanghai, China). Two groups of 8 mice each were given intravenous tail vein injections of 1×10^6^ shControl-Hep-3B cells and shCLCA4-Hep-3B cells, or 1×10^6^ vector-SMMC-7721 cells and CLCA4- SMMC-7721 cells, respectively. After 8 weeks, the mice were sacrificed, and the tumor nodules formed on the lung surfaces were counted. Lungs were excised and embedded in paraffin for further study.

### Follow-up

The last follow-up date was on 31 January 2018. In all the HCC patients (22 females and 164 males), the median follow-up period was 48.5 months (ranging 3.0 - 73.0 months). All patients were conducted once in every 3 months in the first 2 year, once in every 6 months from 2 to 5 year. The follow-up protocol included physical examination, serum alpha-fetoprotein (AFP) level and abdominal ultrasonography. Computed tomography and/or magnetic resonance imaging and/or positron emission tomography were performed when intrahepatic relapse or distant metastasis was suspected. During the follow-up period, there were 101 (54.3%) patients who suffered recurrence and 86 patients (46.2%) died of cancer-related causes. One hundred patients were still alive at the time of the last follow-up report.

### Statistical analysis

All statistical analyses were carried out using SPSS 17.0 statistical software (SPSS Inc, Chicago, IL). The correlation between the expression of CLCA4 and clinicopathologic features was analyzed by χ2 test. Pearson χ2 test was applied to analyze the relationship of CLCA4 expression with EMT and PI3K/AKT signaling markers. Multivariate analysis was performed using the Cox proportional hazards model. Kaplan-Meier analysis and log-rank tests were also performed to compare prognostic differences between two groups. *P* values < 0.05 were considered statistically significant.

## Supplementary Material

Supplementary Figures

Supplementary Table
