# Supplementary material for: CLCA4 inhibits cell proliferation and invasion of hepatocellular carcinoma by suppressing epithelial-mesenchymal transition via PI3K/AKT signaling
Source: Aging (Albany NY). 2018 Oct 11;10(10):2570–84. doi: 10.18632/aging.101571 (PMC6224236; doi:10.18632/aging.101571)
Supplement: Supplementary Figures [file aging-10-101571-s001.pdf]

SUPPLEMENTARY FIGURES

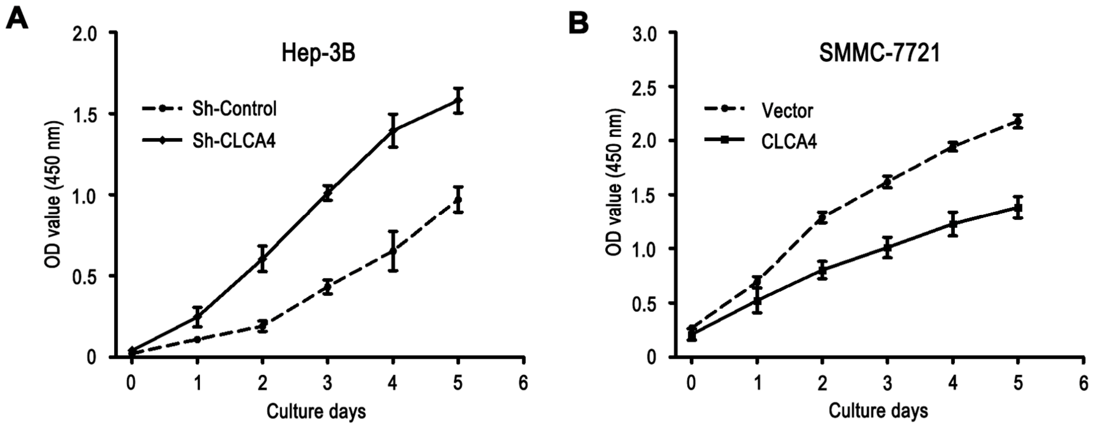

Supplementary Figure S1. CLCA4 inhibits cell proliferation in HCC cells. The cell viability was improved in CLCA4-silenced cells compared with the control cells (**A**), while the opposite effect of cell viability was observed in CLCA4-transfected cells (**B**).

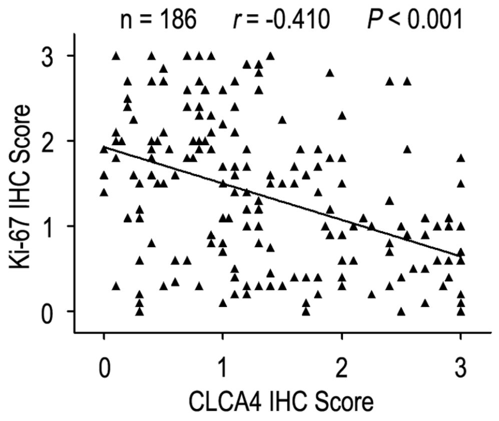

Supplementary Figure S2. The correlation of CLCA4 and Ki-67 expression in HCC tissues. IHC showed CLCA4 expression was negatively correlated with Ki-67 expression.
