# Supplementary material for: CLCA4 inhibits cell proliferation and invasion of hepatocellular carcinoma by suppressing epithelial-mesenchymal transition via PI3K/AKT signaling
Source: Aging (Albany NY). 2018 Oct 11;10(10):2570–84. doi: 10.18632/aging.101571 (PMC6224236; doi:10.18632/aging.101571)
Supplement: Supplementary Table [file aging-10-101571-s002.pdf]

SUPPLEMENTARY TABLE

Supplementary Table S1. Patient characteristics.

Variable	No. of patients (%)
No. of patients	186 (100)
Age: Median [range], y	48 [18-78]
Gender	
Female	22 (11.8)
Male	164 (88.2)
HBsAg	
Negative	12 (6.5)
Positive	174 (93.5)
AFP: Median [range], ng/mL	148.8 [0.6-121000]
GGT: Median [range], U/l	42.6 [3.5-655.5]
Tumor size: Median [range], cm	4.7 [1.0-18.0]
Liver cirrhosis	
No	42 (22.6)
Yes	144 (77.4)
Tumor number	
Single	174 (93.5)
Multiple	12 (6.5)
Satellite nodule	
No	163 (87.6)
Yes	23 (12.4)
Tumor differentiation	
I	9 (4.8)
II	119 (64.0)
III	49 (26.2)
IV	9 (4.8)
Vascular invasion	
No	168 (90.3)
Yes	18 (9.7)
TNM stage	
I	147 (79.0)
II	7 (3.8)
III	32 (17.2)
BCLC stage	
0	17 (9.1)
A	84 (45.2)
B	65 (34.9)
C	20 (10.8)
